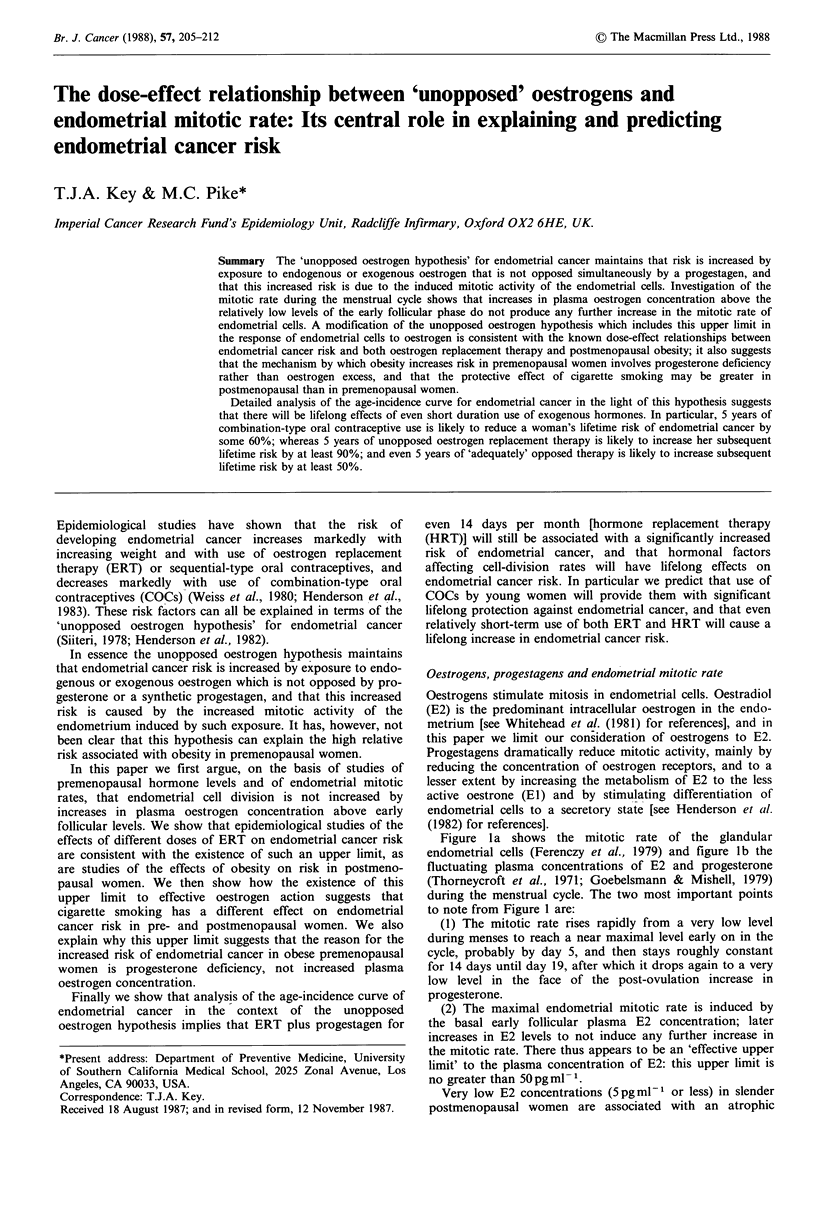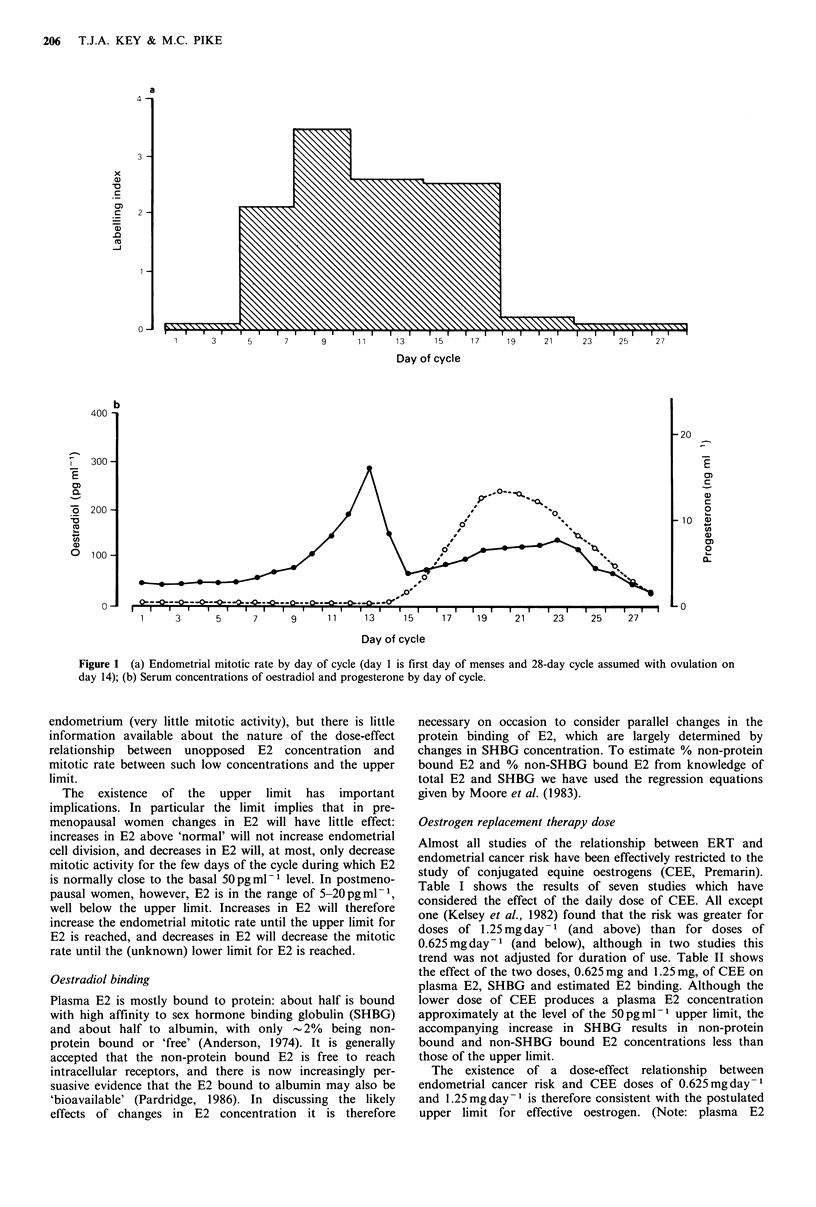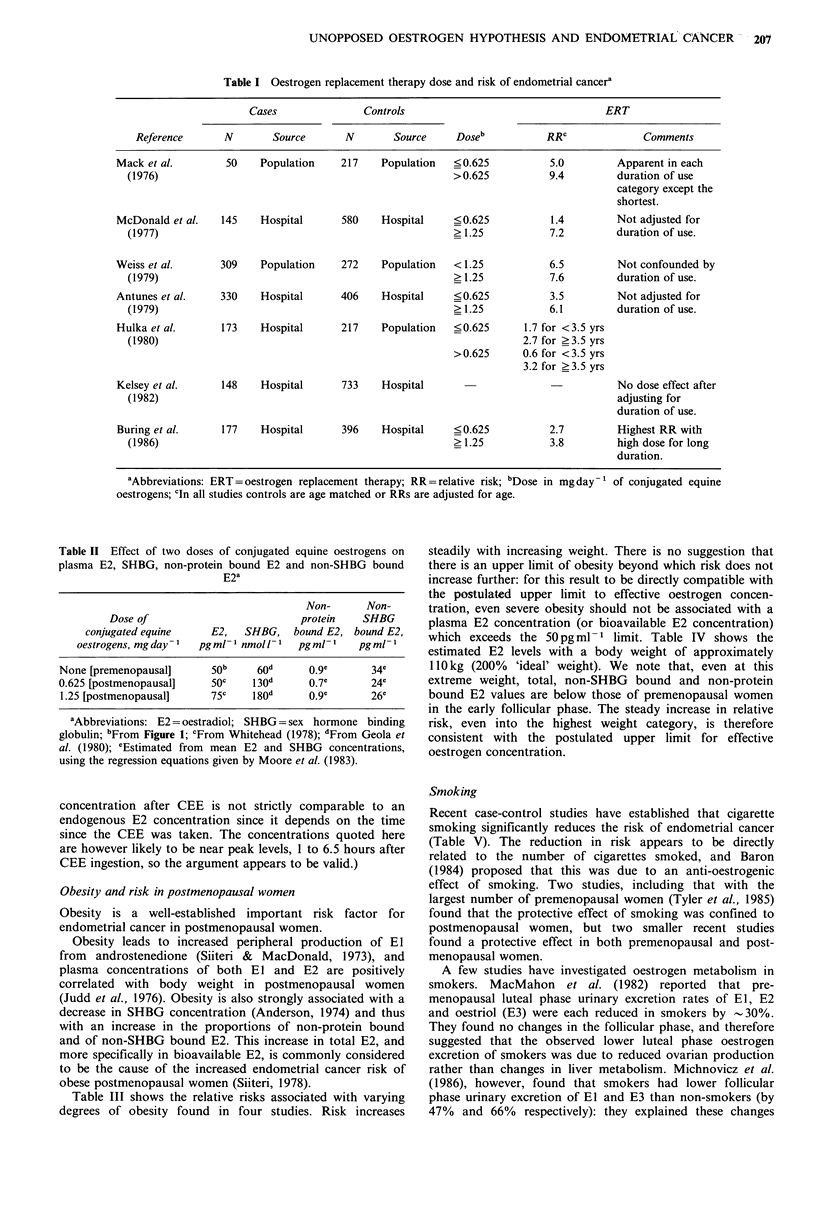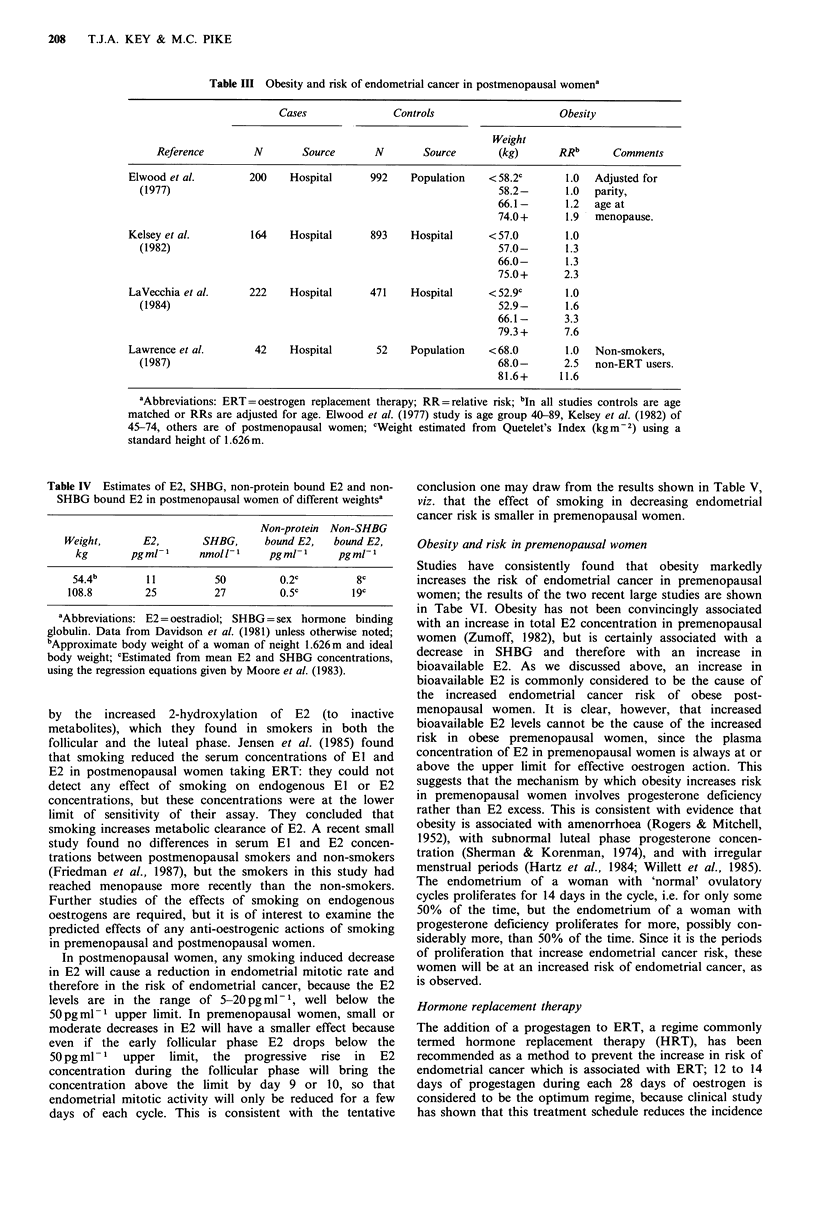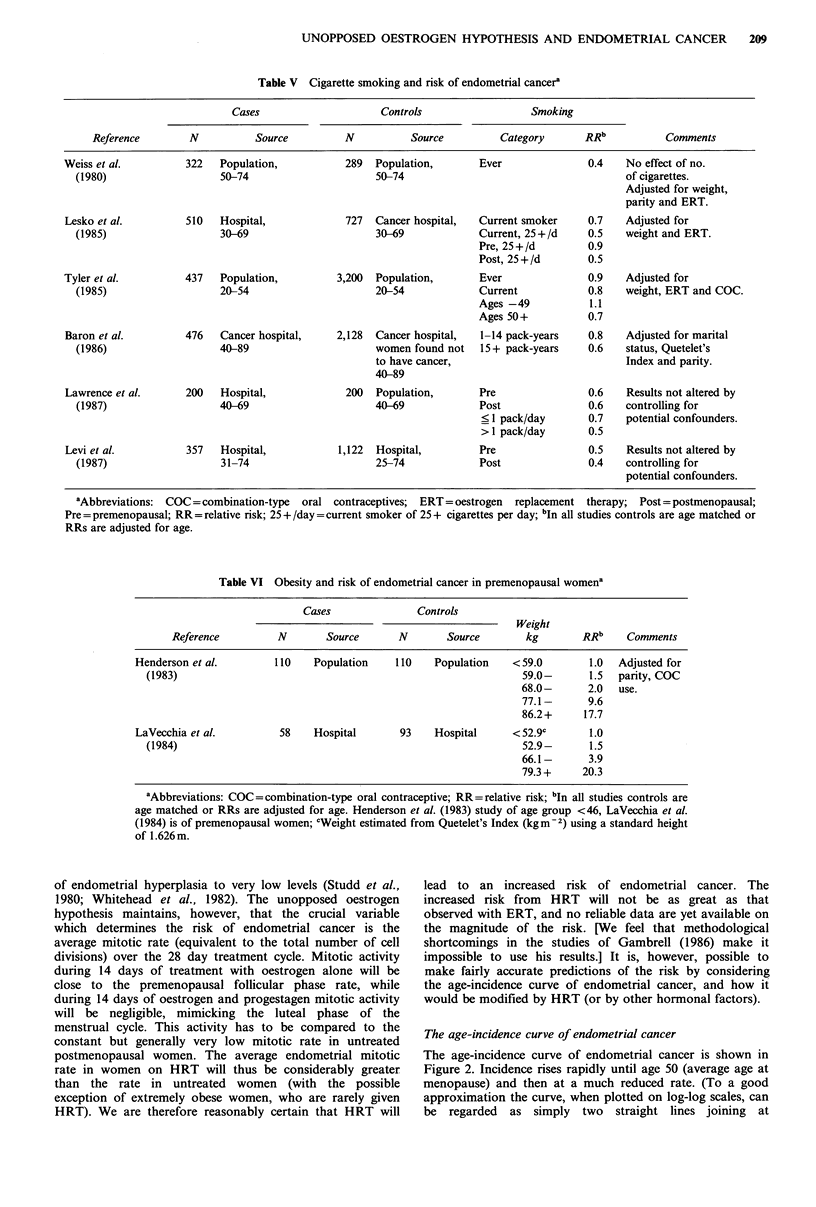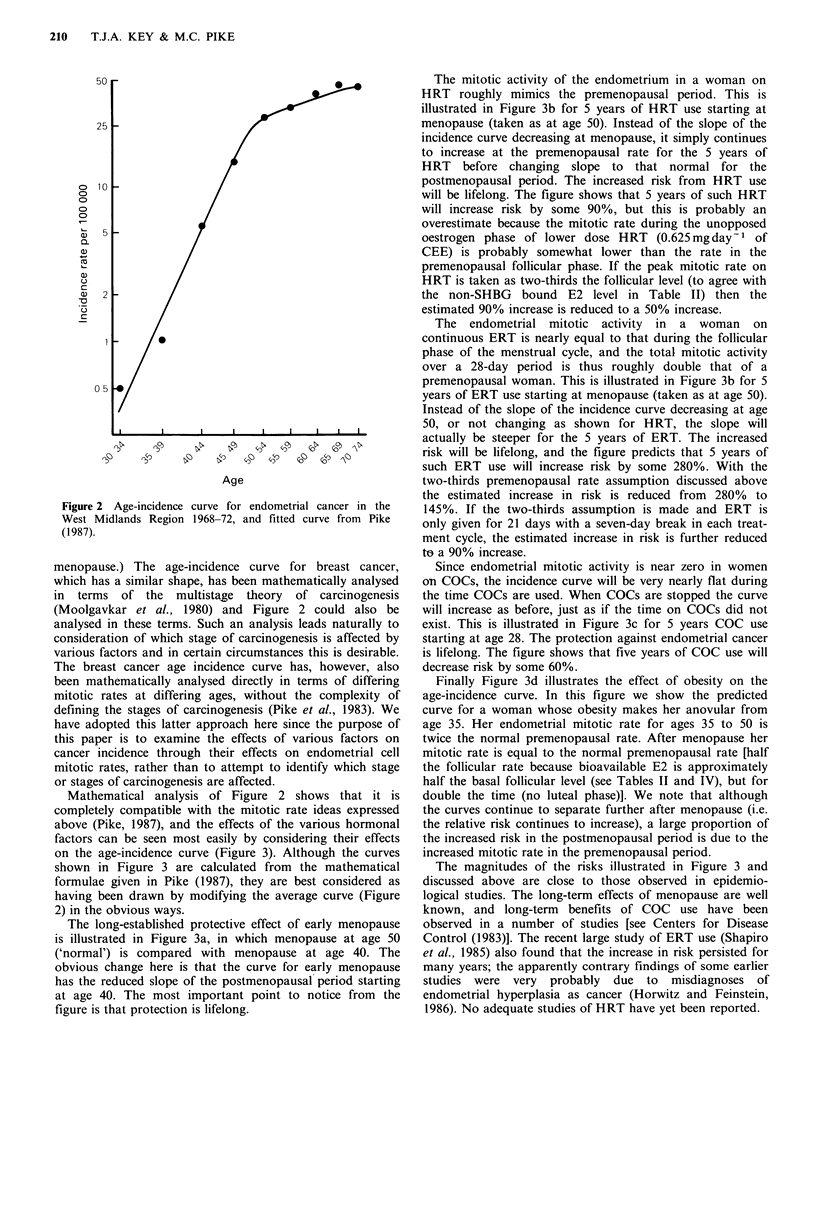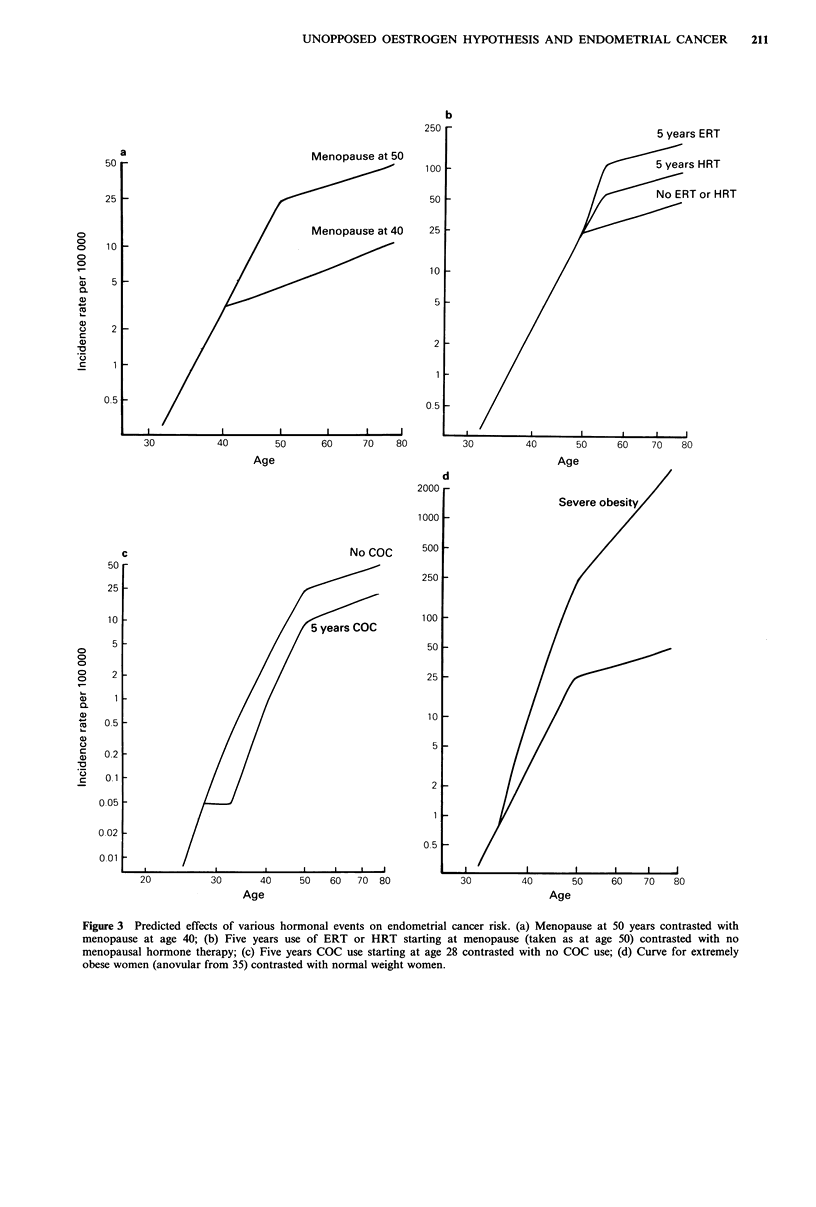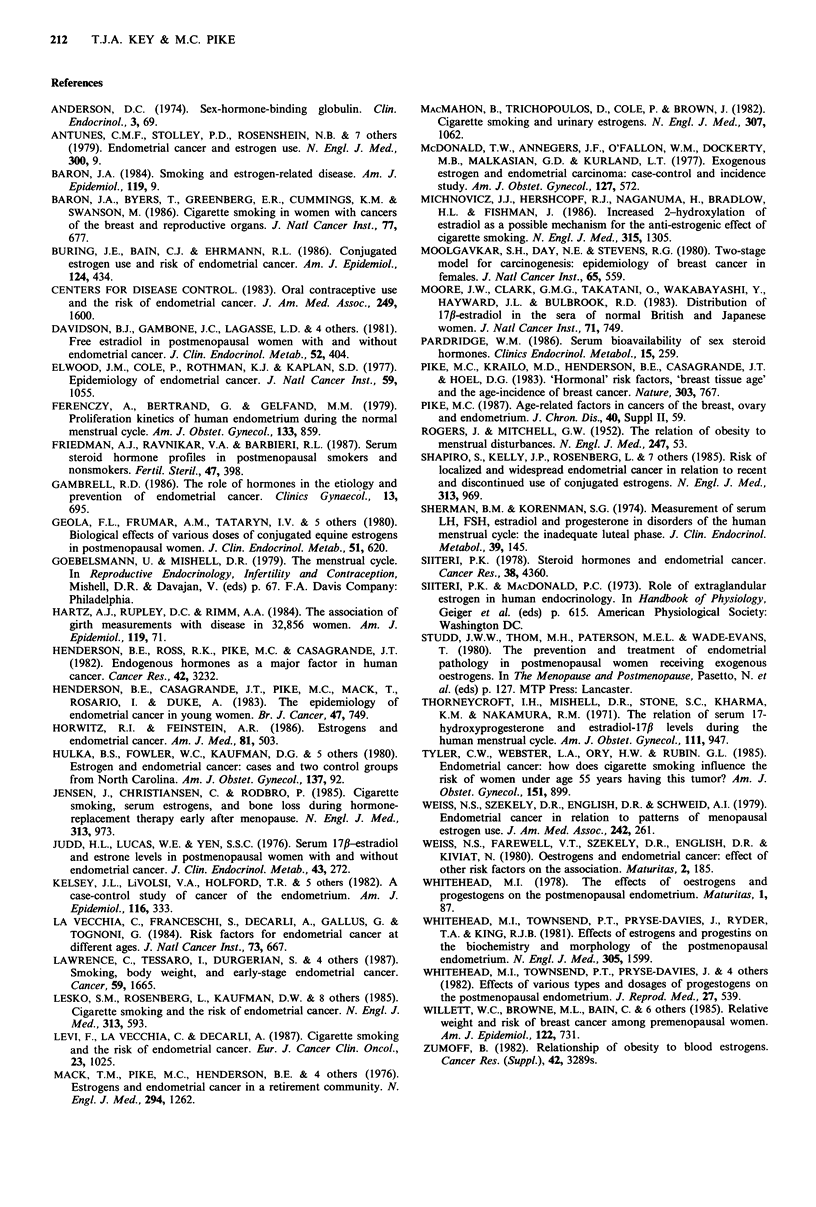# The dose-effect relationship between 'unopposed' oestrogens and endometrial mitotic rate: its central role in explaining and predicting endometrial cancer risk.

**DOI:** 10.1038/bjc.1988.44

**Published:** 1988-02

**Authors:** T. J. Key, M. C. Pike

**Affiliations:** Imperial Cancer Research Fund's Epidemiology Unit, Radcliffe Infirmary, Oxford, UK.

## Abstract

The 'unopposed oestrogen hypothesis' for endometrial cancer maintains that risk is increased by exposure to endogenous or exogenous oestrogen that is not opposed simultaneously by a progestagen, and that this increased risk is due to the induced mitotic activity of the endometrial cells. Investigation of the mitotic rate during the menstrual cycle shows that increases in plasma oestrogen concentration above the relatively low levels of the early follicular phase do not produce any further increase in the mitotic rate of endometrial cells. A modification of the unopposed oestrogen hypothesis which includes this upper limit in the response of endometrial cells to oestrogen is consistent with the known dose-effect relationships between endometrial cancer risk and both oestrogen replacement therapy and postmenopausal obesity; it also suggests that the mechanism by which obesity increases risk in premenopausal women involves progesterone deficiency rather than oestrogen excess, and that the protective effect of cigarette smoking may be greater in postmenopausal than in premenopausal women. Detailed analysis of the age-incidence curve for endometrial cancer in the light of this hypothesis suggests that there will be lifelong effects of even short duration use of exogenous hormones. In particular, 5 years of combination-type oral contraceptive use is likely to reduce a woman's lifetime risk of endometrial cancer by some 60%; whereas 5 years of unopposed oestrogen replacement therapy is likely to increase her subsequent lifetime risk by at least 90%; and even 5 years of 'adequately' opposed therapy is likely to increase subsequent lifetime risk by at least 50%.


*Br. J. Cancer* (1988), **57**, 205–212 © The Macmillan Press Ltd., 1988

### The dose-effect relationship between 'unopposed' oestrogens and endometrial mitotic rate: Its central role in explaining and predicting endometrial cancer risk

T.J.A. Key & M.C. Pike*

*Imperial Cancer Research Fund's Epidemiology Unit, Radcliffe Infirmary, Oxford OX2 6HE, UK.*

**Summary** The 'unopposed oestrogen hypothesis' for endometrial cancer maintains that risk is increased by exposure to endogenous or exogenous oestrogen that is not opposed simultaneously by a progestagen, and that this increased risk is due to the induced mitotic activity of the endometrial cells. Investigation of the mitotic rate during the menstrual cycle shows that increases in plasma oestrogen concentration above the relatively low levels of the early follicular phase do not produce any further increase in the mitotic rate of endometrial cells. A modification of the unopposed oestrogen hypothesis which includes this upper limit in the response of endometrial cells to oestrogen is consistent with the known dose-effect relationships between endometrial cancer risk and both oestrogen replacement therapy and postmenopausal obesity; it also suggests that the mechanism by which obesity increases risk in premenopausal women involves progesterone deficiency rather than oestrogen excess, and that the protective effect of cigarette smoking may be greater in postmenopausal than in premenopausal women.

Detailed analysis of the age-incidence curve for endometrial cancer in the light of this hypothesis suggests that there will be lifelong effects of even short duration use of exogenous hormones. In particular, 5 years of combination-type oral contraceptive use is likely to reduce a woman's lifetime risk of endometrial cancer by some 60%; whereas 5 years of unopposed oestrogen replacement therapy is likely to increase her subsequent lifetime risk by at least 90%; and even 5 years of 'adequately' opposed therapy is likely to increase subsequent lifetime risk by at least 50%.

Epidemiological studies have shown that the risk of developing endometrial cancer increases markedly with increasing weight and with use of oestrogen replacement therapy (ERT) or sequential-type oral contraceptives, and decreases markedly with use of combination-type oral contraceptives (COCs) (Weiss *et al.*, 1980; Henderson *et al.*, 1983). These risk factors can all be explained in terms of the 'unopposed oestrogen hypothesis' for endometrial cancer (Siiteri, 1978; Henderson *et al.*, 1982).

In essence the unopposed oestrogen hypothesis maintains that endometrial cancer risk is increased by exposure to endogenous or exogenous oestrogen which is not opposed by progesterone or a synthetic progestagen, and that this increased risk is caused by the increased mitotic activity of the endometrium induced by such exposure. It has, however, not been clear that this hypothesis can explain the high relative risk associated with obesity in premenopausal women.

In this paper we first argue, on the basis of studies of premenopausal hormone levels and of endometrial mitotic rates, that endometrial cell division is not increased by increases in plasma oestrogen concentration above early follicular levels. We show that epidemiological studies of the effects of different doses of ERT on endometrial cancer risk are consistent with the existence of such an upper limit, as are studies of the effects of obesity on risk in postmenopausal women. We then show how the existence of this upper limit to effective oestrogen action suggests that cigarette smoking has a different effect on endometrial cancer risk in pre- and postmenopausal women. We also explain why this upper limit suggests that the reason for the increased risk of endometrial cancer in obese premenopausal women is progesterone deficiency, not increased plasma oestrogen concentration.

Finally we show that analysis of the age-incidence curve of endometrial cancer in the context of the unopposed oestrogen hypothesis implies that ERT plus progestagen for even 14 days per month [hormone replacement therapy (HRT)] will still be associated with a significantly increased risk of endometrial cancer, and that hormonal factors affecting cell-division rates will have lifelong effects on endometrial cancer risk. In particular we predict that use of COCs by young women will provide them with significant lifelong protection against endometrial cancer, and that even relatively short-term use of both ERT and HRT will cause a lifelong increase in endometrial cancer risk.

*Present address: Department of Preventive Medicine, University of Southern California Medical School, 2025 Zonal Avenue, Los Angeles, CA 90033, USA.
Correspondence: T.J.A. Key.
Received 18 August 1987; and in revised form, 12 November 1987.

### *Oestrogens, progestagens and endometrial mitotic rate*

Oestrogens stimulate mitosis in endometrial cells. Oestradiol (E2) is the predominant intracellular oestrogen in the endometrium [see Whitehead *et al.* (1981) for references], and in this paper we limit our consideration of oestrogens to E2. Progestagens dramatically reduce mitotic activity, mainly by reducing the concentration of oestrogen receptors, and to a lesser extent by increasing the metabolism of E2 to the less active oestrone (E1) and by stimulating differentiation of endometrial cells to a secretory state [see Henderson *et al.* (1982) for references].

Figure 1a shows the mitotic rate of the glandular endometrial cells (Ferenczy *et al.*, 1979) and figure 1b the fluctuating plasma concentrations of E2 and progesterone (Thorneycroft *et al.*, 1971; Goebelsmann & Mishell, 1979) during the menstrual cycle. The two most important points to note from Figure 1 are:

(1) The mitotic rate rises rapidly from a very low level during menses to reach a near maximal level early on in the cycle, probably by day 5, and then stays roughly constant for 14 days until day 19, after which it drops again to a very low level in the face of the post-ovulation increase in progesterone.

(2) The maximal endometrial mitotic rate is induced by the basal early follicular plasma E2 concentration; later increases in E2 levels to not induce any further increase in the mitotic rate. There thus appears to be an 'effective upper limit' to the plasma concentration of E2: this upper limit is no greater than $50\,pg\,ml^{-1}$.

Very low E2 concentrations ($5\,pg\,ml^{-1}$ or less) in slender postmenopausal women are associated with an atrophic

**Figure 1** (a) Endometrial mitotic rate by day of cycle (day 1 is first day of menses and 28-day cycle assumed with ovulation on day 14); (b) Serum concentrations of oestradiol and progesterone by day of cycle.

endometrium (very little mitotic activity), but there is little information available about the nature of the dose-effect relationship between unopposed E2 concentration and mitotic rate between such low concentrations and the upper limit.

The existence of the upper limit has important implications. In particular the limit implies that in premenopausal women changes in E2 will have little effect: increases in E2 above 'normal' will not increase endometrial cell division, and decreases in E2 will, at most, only decrease mitotic activity for the few days of the cycle during which E2 is normally close to the basal 50 pg ml$^{-1}$ level. In postmenopausal women, however, E2 is in the range of 5–20 pg ml$^{-1}$, well below the upper limit. Increases in E2 will therefore increase the endometrial mitotic rate until the upper limit for E2 is reached, and decreases in E2 will decrease the mitotic rate until the (unknown) lower limit for E2 is reached.

*Oestradiol binding*

Plasma E2 is mostly bound to protein: about half is bound with high affinity to sex hormone binding globulin (SHBG) and about half to albumin, with only ~2% being non-protein bound or 'free' (Anderson, 1974). It is generally accepted that the non-protein bound E2 is free to reach intracellular receptors, and there is now increasingly persuasive evidence that the E2 bound to albumin may also be 'bioavailable' (Pardridge, 1986). In discussing the likely effects of changes in E2 concentration it is therefore necessary on occasion to consider parallel changes in the protein binding of E2, which are largely determined by changes in SHBG concentration. To estimate % non-protein bound E2 and % non-SHBG bound E2 from knowledge of total E2 and SHBG we have used the regression equations given by Moore *et al.* (1983).

*Oestrogen replacement therapy dose*

Almost all studies of the relationship between ERT and endometrial cancer risk have been effectively restricted to the study of conjugated equine oestrogens (CEE, Premarin). Table I shows the results of seven studies which have considered the effect of the daily dose of CEE. All except one (Kelsey *et al.*, 1982) found that the risk was greater for doses of 1.25 mg day$^{-1}$ (and above) than for doses of 0.625 mg day$^{-1}$ (and below), although in two studies this trend was not adjusted for duration of use. Table II shows the effect of the two doses, 0.625 mg and 1.25 mg, of CEE on plasma E2, SHBG and estimated E2 binding. Although the lower dose of CEE produces a plasma E2 concentration approximately at the level of the 50 pg ml$^{-1}$ upper limit, the accompanying increase in SHBG results in non-protein bound and non-SHBG bound E2 concentrations less than those of the upper limit.

The existence of a dose-effect relationship between endometrial cancer risk and CEE doses of 0.625 mg day$^{-1}$ and 1.25 mg day$^{-1}$ is therefore consistent with the postulated upper limit for effective oestrogen. (Note: plasma E2

**Table I** Oestrogen replacement therapy dose and risk of endometrial cancer[a]

| | *Cases* | | *Controls* | | | *ERT* | |
|---|---|---|---|---|---|---|---|
| *Reference* | *N* | *Source* | *N* | *Source* | *Dose*[b] | *RR*[c] | *Comments* |
| Mack *et al.* (1976) | 50 | Population | 217 | Population | ≦0.625<br>>0.625 | 5.0<br>9.4 | Apparent in each duration of use category except the shortest. |
| McDonald *et al.* (1977) | 145 | Hospital | 580 | Hospital | ≦0.625<br>≧1.25 | 1.4<br>7.2 | Not adjusted for duration of use. |
| Weiss *et al.* (1979) | 309 | Population | 272 | Population | <1.25<br>≧1.25 | 6.5<br>7.6 | Not confounded by duration of use. |
| Antunes *et al.* (1979) | 330 | Hospital | 406 | Hospital | ≦0.625<br>≧1.25 | 3.5<br>6.1 | Not adjusted for duration of use. |
| Hulka *et al.* (1980) | 173 | Hospital | 217 | Population | ≦0.625<br><br>>0.625 | 1.7 for <3.5 yrs<br>2.7 for ≧3.5 yrs<br>0.6 for <3.5 yrs<br>3.2 for ≧3.5 yrs | |
| Kelsey *et al.* (1982) | 148 | Hospital | 733 | Hospital | — | — | No dose effect after adjusting for duration of use. |
| Buring *et al.* (1986) | 177 | Hospital | 396 | Hospital | ≦0.625<br>≧1.25 | 2.7<br>3.8 | Highest RR with high dose for long duration. |

[a]Abbreviations: ERT = oestrogen replacement therapy; RR = relative risk; [b]Dose in mg day$^{-1}$ of conjugated equine oestrogens; [c]In all studies controls are age matched or RRs are adjusted for age.

**Table II** Effect of two doses of conjugated equine oestrogens on plasma E2, SHBG, non-protein bound E2 and non-SHBG bound E2[a]

| *Dose of conjugated equine oestrogens, mg day*$^{-1}$ | *E2, pg ml*$^{-1}$ | *SHBG, nmol l*$^{-1}$ | *Non-protein bound E2, pg ml*$^{-1}$ | *Non-SHBG bound E2, pg ml*$^{-1}$ |
|---|---|---|---|---|
| None [premenopausal] | 50[b] | 60[d] | 0.9[e] | 34[e] |
| 0.625 [postmenopausal] | 50[c] | 130[d] | 0.7[e] | 24[e] |
| 1.25 [postmenopausal] | 75[c] | 180[d] | 0.9[e] | 26[e] |

[a]Abbreviations: E2 = oestradiol; SHBG = sex hormone binding globulin; [b]From **Figure 1**; [c]From Whitehead (1978); [d]From Geola *et al.* (1980); [e]Estimated from mean E2 and SHBG concentrations, using the regression equations given by Moore *et al.* (1983).

concentration after CEE is not strictly comparable to an endogenous E2 concentration since it depends on the time since the CEE was taken. The concentrations quoted here are however likely to be near peak levels, 1 to 6.5 hours after CEE ingestion, so the argument appears to be valid.)

### *Obesity and risk in postmenopausal women*

Obesity is a well-established important risk factor for endometrial cancer in postmenopausal women.

Obesity leads to increased peripheral production of E1 from androstenedione (Siiteri & MacDonald, 1973), and plasma concentrations of both E1 and E2 are positively correlated with body weight in postmenopausal women (Judd *et al.*, 1976). Obesity is also strongly associated with a decrease in SHBG concentration (Anderson, 1974) and thus with an increase in the proportions of non-protein bound and of non-SHBG bound E2. This increase in total E2, and more specifically in bioavailable E2, is commonly considered to be the cause of the increased endometrial cancer risk of obese postmenopausal women (Siiteri, 1978).

Table III shows the relative risks associated with varying degrees of obesity found in four studies. Risk increases steadily with increasing weight. There is no suggestion that there is an upper limit of obesity beyond which risk does not increase further: for this result to be directly compatible with the postulated upper limit to effective oestrogen concentration, even severe obesity should not be associated with a plasma E2 concentration (or bioavailable E2 concentration) which exceeds the 50 pg ml$^{-1}$ limit. Table IV shows the estimated E2 levels with a body weight of approximately 110 kg (200% 'ideal' weight). We note that, even at this extreme weight, total, non-SHBG bound and non-protein bound E2 values are below those of premenopausal women in the early follicular phase. The steady increase in relative risk, even into the highest weight category, is therefore consistent with the postulated upper limit for effective oestrogen concentration.

### *Smoking*

Recent case-control studies have established that cigarette smoking significantly reduces the risk of endometrial cancer (Table V). The reduction in risk appears to be directly related to the number of cigarettes smoked, and Baron (1984) proposed that this was due to an anti-oestrogenic effect of smoking. Two studies, including that with the largest number of premenopausal women (Tyler *et al.*, 1985) found that the protective effect of smoking was confined to postmenopausal women, but two smaller recent studies found a protective effect in both premenopausal and postmenopausal women.

A few studies have investigated oestrogen metabolism in smokers. MacMahon *et al.* (1982) reported that premenopausal luteal phase urinary excretion rates of E1, E2 and oestriol (E3) were each reduced in smokers by ~30%. They found no changes in the follicular phase, and therefore suggested that the observed lower luteal phase oestrogen excretion of smokers was due to reduced ovarian production rather than changes in liver metabolism. Michnovicz *et al.* (1986), however, found that smokers had lower follicular phase urinary excretion of E1 and E3 than non-smokers (by 47% and 66% respectively): they explained these changes

**Table III** Obesity and risk of endometrial cancer in postmenopausal women[a]

| | Cases | | Controls | | Obesity | | |
|---|---|---|---|---|---|---|---|
| *Reference* | *N* | *Source* | *N* | *Source* | *Weight (kg)* | *RR*[b] | *Comments* |
| Elwood *et al.* (1977) | 200 | Hospital | 992 | Population | <58.2[c] | 1.0 | Adjusted for |
| | | | | | 58.2– | 1.0 | parity, |
| | | | | | 66.1– | 1.2 | age at |
| | | | | | 74.0+ | 1.9 | menopause. |
| Kelsey *et al.* (1982) | 164 | Hospital | 893 | Hospital | <57.0 | 1.0 | |
| | | | | | 57.0– | 1.3 | |
| | | | | | 66.0– | 1.3 | |
| | | | | | 75.0+ | 2.3 | |
| LaVecchia *et al.* (1984) | 222 | Hospital | 471 | Hospital | <52.9[c] | 1.0 | |
| | | | | | 52.9– | 1.6 | |
| | | | | | 66.1– | 3.3 | |
| | | | | | 79.3+ | 7.6 | |
| Lawrence *et al.* (1987) | 42 | Hospital | 52 | Population | <68.0 | 1.0 | Non-smokers, |
| | | | | | 68.0– | 2.5 | non-ERT users. |
| | | | | | 81.6+ | 11.6 | |

[a]Abbreviations: ERT = oestrogen replacement therapy; RR = relative risk; [b]In all studies controls are age matched or RRs are adjusted for age. Elwood *et al.* (1977) study is age group 40–89, Kelsey *et al.* (1982) of 45–74, others are of postmenopausal women; [c]Weight estimated from Quetelet's Index (kg m$^{-2}$) using a standard height of 1.626 m.

**Table IV** Estimates of E2, SHBG, non-protein bound E2 and non-SHBG bound E2 in postmenopausal women of different weights[a]

| *Weight, kg* | *E2, pg ml$^{-1}$* | *SHBG, nmol l$^{-1}$* | *Non-protein bound E2, pg ml$^{-1}$* | *Non-SHBG bound E2, pg ml$^{-1}$* |
|---|---|---|---|---|
| 54.4[b] | 11 | 50 | 0.2[c] | 8[c] |
| 108.8 | 25 | 27 | 0.5[c] | 19[c] |

[a]Abbreviations: E2 = oestradiol; SHBG = sex hormone binding globulin. Data from Davidson *et al.* (1981) unless otherwise noted; [b]Approximate body weight of a woman of neight 1.626 m and ideal body weight; [c]Estimated from mean E2 and SHBG concentrations, using the regression equations given by Moore *et al.* (1983).

by the increased 2-hydroxylation of E2 (to inactive metabolites), which they found in smokers in both the follicular and the luteal phase. Jensen *et al.* (1985) found that smoking reduced the serum concentrations of E1 and E2 in postmenopausal women taking ERT: they could not detect any effect of smoking on endogenous E1 or E2 concentrations, but these concentrations were at the lower limit of sensitivity of their assay. They concluded that smoking increases metabolic clearance of E2. A recent small study found no differences in serum E1 and E2 concentrations between postmenopausal smokers and non-smokers (Friedman *et al.*, 1987), but the smokers in this study had reached menopause more recently than the non-smokers. Further studies of the effects of smoking on endogenous oestrogens are required, but it is of interest to examine the predicted effects of any anti-oestrogenic actions of smoking in premenopausal and postmenopausal women.

In postmenopausal women, any smoking induced decrease in E2 will cause a reduction in endometrial mitotic rate and therefore in the risk of endometrial cancer, because the E2 levels are in the range of 5–20 pg ml$^{-1}$, well below the 50 pg ml$^{-1}$ upper limit. In premenopausal women, small or moderate decreases in E2 will have a smaller effect because even if the early follicular phase E2 drops below the 50 pg ml$^{-1}$ upper limit, the progressive rise in E2 concentration during the follicular phase will bring the concentration above the limit by day 9 or 10, so that endometrial mitotic activity will only be reduced for a few days of each cycle. This is consistent with the tentative conclusion one may draw from the results shown in Table V, *viz.* that the effect of smoking in decreasing endometrial cancer risk is smaller in premenopausal women.

*Obesity and risk in premenopausal women*

Studies have consistently found that obesity markedly increases the risk of endometrial cancer in premenopausal women; the results of the two recent large studies are shown in Tabe VI. Obesity has not been convincingly associated with an increase in total E2 concentration in premenopausal women (Zumoff, 1982), but is certainly associated with a decrease in SHBG and therefore with an increase in bioavailable E2. As we discussed above, an increase in bioavailable E2 is commonly considered to be the cause of the increased endometrial cancer risk of obese postmenopausal women. It is clear, however, that increased bioavailable E2 levels cannot be the cause of the increased risk in obese premenopausal women, since the plasma concentration of E2 in premenopausal women is always at or above the upper limit for effective oestrogen action. This suggests that the mechanism by which obesity increases risk in premenopausal women involves progesterone deficiency rather than E2 excess. This is consistent with evidence that obesity is associated with amenorrhoea (Rogers & Mitchell, 1952), with subnormal luteal phase progesterone concentration (Sherman & Korenman, 1974), and with irregular menstrual periods (Hartz *et al.*, 1984; Willett *et al.*, 1985). The endometrium of a woman with 'normal' ovulatory cycles proliferates for 14 days in the cycle, i.e. for only some 50% of the time, but the endometrium of a woman with progesterone deficiency proliferates for more, possibly considerably more, than 50% of the time. Since it is the periods of proliferation that increase endometrial cancer risk, these women will be at an increased risk of endometrial cancer, as is observed.

*Hormone replacement therapy*

The addition of a progestagen to ERT, a regime commonly termed hormone replacement therapy (HRT), has been recommended as a method to prevent the increase in risk of endometrial cancer which is associated with ERT; 12 to 14 days of progestagen during each 28 days of oestrogen is considered to be the optimum regime, because clinical study has shown that this treatment schedule reduces the incidence

**Table V** Cigarette smoking and risk of endometrial cancer[a]

| Reference | Cases N | Cases Source | Controls N | Controls Source | Smoking Category | Smoking RR[b] | Comments |
|---|---|---|---|---|---|---|---|
| Weiss *et al.* (1980) | 322 | Population, 50–74 | 289 | Population, 50–74 | Ever | 0.4 | No effect of no. of cigarettes. Adjusted for weight, parity and ERT. |
| Lesko *et al.* (1985) | 510 | Hospital, 30–69 | 727 | Cancer hospital, 30–69 | Current smoker<br>Current, 25+/d<br>Pre, 25+/d<br>Post, 25+/d | 0.7<br>0.5<br>0.9<br>0.5 | Adjusted for weight and ERT. |
| Tyler *et al.* (1985) | 437 | Population, 20–54 | 3,200 | Population, 20–54 | Ever<br>Current<br>Ages –49<br>Ages 50+ | 0.9<br>0.8<br>1.1<br>0.7 | Adjusted for weight, ERT and COC. |
| Baron *et al.* (1986) | 476 | Cancer hospital, 40–89 | 2,128 | Cancer hospital, women found not to have cancer, 40–89 | 1–14 pack-years<br>15+ pack-years | 0.8<br>0.6 | Adjusted for marital status, Quetelet's Index and parity. |
| Lawrence *et al.* (1987) | 200 | Hospital, 40–69 | 200 | Population, 40–69 | Pre<br>Post<br>≦1 pack/day<br>>1 pack/day | 0.6<br>0.6<br>0.7<br>0.5 | Results not altered by controlling for potential confounders. |
| Levi *et al.* (1987) | 357 | Hospital, 31–74 | 1,122 | Hospital, 25–74 | Pre<br>Post | 0.5<br>0.4 | Results not altered by controlling for potential confounders. |

[a]Abbreviations: COC = combination-type oral contraceptives; ERT = oestrogen replacement therapy; Post = postmenopausal; Pre = premenopausal; RR = relative risk; 25+/day = current smoker of 25+ cigarettes per day; [b]In all studies controls are age matched or RRs are adjusted for age.

**Table VI** Obesity and risk of endometrial cancer in premenopausal women[a]

| Reference | Cases N | Cases Source | Controls N | Controls Source | Weight kg | RR[b] | Comments |
|---|---|---|---|---|---|---|---|
| Henderson *et al.* (1983) | 110 | Population | 110 | Population | <59.0<br>59.0–<br>68.0–<br>77.1–<br>86.2+ | 1.0<br>1.5<br>2.0<br>9.6<br>17.7 | Adjusted for parity, COC use. |
| LaVecchia *et al.* (1984) | 58 | Hospital | 93 | Hospital | <52.9[c]<br>52.9–<br>66.1–<br>79.3+ | 1.0<br>1.5<br>3.9<br>20.3 | |

[a]Abbreviations: COC = combination-type oral contraceptive; RR = relative risk; [b]In all studies controls are age matched or RRs are adjusted for age. Henderson *et al.* (1983) study of age group <46, LaVecchia *et al.* (1984) is of premenopausal women; [c]Weight estimated from Quetelet's Index ($kg\,m^{-2}$) using a standard height of 1.626 m.

of endometrial hyperplasia to very low levels (Studd *et al.*, 1980; Whitehead *et al.*, 1982). The unopposed oestrogen hypothesis maintains, however, that the crucial variable which determines the risk of endometrial cancer is the average mitotic rate (equivalent to the total number of cell divisions) over the 28 day treatment cycle. Mitotic activity during 14 days of treatment with oestrogen alone will be close to the premenopausal follicular phase rate, while during 14 days of oestrogen and progestagen mitotic activity will be negligible, mimicking the luteal phase of the menstrual cycle. This activity has to be compared to the constant but generally very low mitotic rate in untreated postmenopausal women. The average endometrial mitotic rate in women on HRT will thus be considerably greater than the rate in untreated women (with the possible exception of extremely obese women, who are rarely given HRT). We are therefore reasonably certain that HRT will lead to an increased risk of endometrial cancer. The increased risk from HRT will not be as great as that observed with ERT, and no reliable data are yet available on the magnitude of the risk. [We feel that methodological shortcomings in the studies of Gambrell (1986) make it impossible to use his results.] It is, however, possible to make fairly accurate predictions of the risk by considering the age-incidence curve of endometrial cancer, and how it would be modified by HRT (or by other hormonal factors).

*The age-incidence curve of endometrial cancer*

The age-incidence curve of endometrial cancer is shown in Figure 2. Incidence rises rapidly until age 50 (average age at menopause) and then at a much reduced rate. (To a good approximation the curve, when plotted on log-log scales, can be regarded as simply two straight lines joining at

**Figure 2** Age-incidence curve for endometrial cancer in the West Midlands Region 1968–72, and fitted curve from Pike (1987).

menopause.) The age-incidence curve for breast cancer, which has a similar shape, has been mathematically analysed in terms of the multistage theory of carcinogenesis (Moolgavkar *et al.*, 1980) and Figure 2 could also be analysed in these terms. Such an analysis leads naturally to consideration of which stage of carcinogenesis is affected by various factors and in certain circumstances this is desirable. The breast cancer age incidence curve has, however, also been mathematically analysed directly in terms of differing mitotic rates at differing ages, without the complexity of defining the stages of carcinogenesis (Pike *et al.*, 1983). We have adopted this latter approach here since the purpose of this paper is to examine the effects of various factors on cancer incidence through their effects on endometrial cell mitotic rates, rather than to attempt to identify which stage or stages of carcinogenesis are affected.

Mathematical analysis of Figure 2 shows that it is completely compatible with the mitotic rate ideas expressed above (Pike, 1987), and the effects of the various hormonal factors can be seen most easily by considering their effects on the age-incidence curve (Figure 3). Although the curves shown in Figure 3 are calculated from the mathematical formulae given in Pike (1987), they are best considered as having been drawn by modifying the average curve (Figure 2) in the obvious ways.

The long-established protective effect of early menopause is illustrated in Figure 3a, in which menopause at age 50 ('normal') is compared with menopause at age 40. The obvious change here is that the curve for early menopause has the reduced slope of the postmenopausal period starting at age 40. The most important point to notice from the figure is that protection is lifelong.

The mitotic activity of the endometrium in a woman on HRT roughly mimics the premenopausal period. This is illustrated in Figure 3b for 5 years of HRT use starting at menopause (taken as at age 50). Instead of the slope of the incidence curve decreasing at menopause, it simply continues to increase at the premenopausal rate for the 5 years of HRT before changing slope to that normal for the postmenopausal period. The increased risk from HRT use will be lifelong. The figure shows that 5 years of such HRT will increase risk by some 90%, but this is probably an overestimate because the mitotic rate during the unopposed oestrogen phase of lower dose HRT (0.625 mg day$^{-1}$ of CEE) is probably somewhat lower than the rate in the premenopausal follicular phase. If the peak mitotic rate on HRT is taken as two-thirds the follicular level (to agree with the non-SHBG bound E2 level in Table II) then the estimated 90% increase is reduced to a 50% increase.

The endometrial mitotic activity in a woman on continuous ERT is nearly equal to that during the follicular phase of the menstrual cycle, and the total mitotic activity over a 28-day period is thus roughly double that of a premenopausal woman. This is illustrated in Figure 3b for 5 years of ERT use starting at menopause (taken as at age 50). Instead of the slope of the incidence curve decreasing at age 50, or not changing as shown for HRT, the slope will actually be steeper for the 5 years of ERT. The increased risk will be lifelong, and the figure predicts that 5 years of such ERT use will increase risk by some 280%. With the two-thirds premenopausal rate assumption discussed above the estimated increase in risk is reduced from 280% to 145%. If the two-thirds assumption is made and ERT is only given for 21 days with a seven-day break in each treatment cycle, the estimated increase in risk is further reduced to a 90% increase.

Since endometrial mitotic activity is near zero in women on COCs, the incidence curve will be very nearly flat during the time COCs are used. When COCs are stopped the curve will increase as before, just as if the time on COCs did not exist. This is illustrated in Figure 3c for 5 years COC use starting at age 28. The protection against endometrial cancer is lifelong. The figure shows that five years of COC use will decrease risk by some 60%.

Finally Figure 3d illustrates the effect of obesity on the age-incidence curve. In this figure we show the predicted curve for a woman whose obesity makes her anovular from age 35. Her endometrial mitotic rate for ages 35 to 50 is twice the normal premenopausal rate. After menopause her mitotic rate is equal to the normal premenopausal rate [half the follicular rate because bioavailable E2 is approximately half the basal follicular level (see Tables II and IV), but for double the time (no luteal phase)]. We note that although the curves continue to separate further after menopause (i.e. the relative risk continues to increase), a large proportion of the increased risk in the postmenopausal period is due to the increased mitotic rate in the premenopausal period.

The magnitudes of the risks illustrated in Figure 3 and discussed above are close to those observed in epidemiological studies. The long-term effects of menopause are well known, and long-term benefits of COC use have been observed in a number of studies [see Centers for Disease Control (1983)]. The recent large study of ERT use (Shapiro *et al.*, 1985) also found that the increase in risk persisted for many years; the apparently contrary findings of some earlier studies were very probably due to misdiagnoses of endometrial hyperplasia as cancer (Horwitz and Feinstein, 1986). No adequate studies of HRT have yet been reported.

**Figure 3** Predicted effects of various hormonal events on endometrial cancer risk. (a) Menopause at 50 years contrasted with menopause at age 40; (b) Five years use of ERT or HRT starting at menopause (taken as at age 50) contrasted with no menopausal hormone therapy; (c) Five years COC use starting at age 28 contrasted with no COC use; (d) Curve for extremely obese women (anovular from 35) contrasted with normal weight women.

### References

ANDERSON, D.C. (1974). Sex-hormone-binding globulin. *Clin. Endocrinol.*, **3**, 69.

ANTUNES, C.M.F., STOLLEY, P.D., ROSENSHEIN, N.B. & 7 others (1979). Endometrial cancer and estrogen use. *N. Engl. J. Med.*, **300**, 9.

BARON, J.A. (1984). Smoking and estrogen-related disease. *Am. J. Epidemiol.*, **119**, 9.

BARON, J.A., BYERS, T., GREENBERG, E.R., CUMMINGS, K.M. & SWANSON, M. (1986). Cigarette smoking in women with cancers of the breast and reproductive organs. *J. Natl Cancer Inst.*, **77**, 677.

BURING, J.E., BAIN, C.J. & EHRMANN, R.L. (1986). Conjugated estrogen use and risk of endometrial cancer. *Am. J. Epidemiol.*, **124**, 434.

CENTERS FOR DISEASE CONTROL. (1983). Oral contraceptive use and the risk of endometrial cancer. *J. Am. Med. Assoc.*, **249**, 1600.

DAVIDSON, B.J., GAMBONE, J.C., LAGASSE, L.D. & 4 others. (1981). Free estradiol in postmenopausal women with and without endometrial cancer. *J. Clin. Endocrinol. Metab.*, **52**, 404.

ELWOOD, J.M., COLE, P., ROTHMAN, K.J. & KAPLAN, S.D. (1977). Epidemiology of endometrial cancer. *J. Natl Cancer Inst.*, **59**, 1055.

FERENCZY, A., BERTRAND, G. & GELFAND, M.M. (1979). Proliferation kinetics of human endometrium during the normal menstrual cycle. *Am. J. Obstet. Gynecol.*, **133**, 859.

FRIEDMAN, A.J., RAVNIKAR, V.A. & BARBIERI, R.L. (1987). Serum steroid hormone profiles in postmenopausal smokers and nonsmokers. *Fertil. Steril.*, **47**, 398.

GAMBRELL, R.D. (1986). The role of hormones in the etiology and prevention of endometrial cancer. *Clinics Gynaecol.*, **13**, 695.

GEOLA, F.L., FRUMAR, A.M., TATARYN, I.V. & 5 others (1980). Biological effects of various doses of conjugated equine estrogens in postmenopausal women. *J. Clin. Endocrinol. Metab.*, **51**, 620.

GOEBELSMANN, U. & MISHELL, D.R. (1979). The menstrual cycle. In *Reproductive Endocrinology, Infertility and Contraception*, Mishell, D.R. & Davajan, V. (eds) p. 67. F.A. Davis Company: Philadelphia.

HARTZ, A.J., RUPLEY, D.C. & RIMM, A.A. (1984). The association of girth measurements with disease in 32,856 women. *Am. J. Epidemiol.*, **119**, 71.

HENDERSON, B.E., ROSS, R.K., PIKE, M.C. & CASAGRANDE, J.T. (1982). Endogenous hormones as a major factor in human cancer. *Cancer Res.*, **42**, 3232.

HENDERSON, B.E., CASAGRANDE, J.T., PIKE, M.C., MACK, T., ROSARIO, I. & DUKE, A. (1983). The epidemiology of endometrial cancer in young women. *Br. J. Cancer*, **47**, 749.

HORWITZ, R.I. & FEINSTEIN, A.R. (1986). Estrogens and endometrial cancer. *Am. J. Med.*, **81**, 503.

HULKA, B.S., FOWLER, W.C., KAUFMAN, D.G. & 5 others (1980). Estrogen and endometrial cancer: cases and two control groups from North Carolina. *Am. J. Obstet. Gynecol.*, **137**, 92.

JENSEN, J., CHRISTIANSEN, C. & RODBRO, P. (1985). Cigarette smoking, serum estrogens, and bone loss during hormone-replacement therapy early after menopause. *N. Engl. J. Med.*, **313**, 973.

JUDD, H.L., LUCAS, W.E. & YEN, S.S.C. (1976). Serum 17$\beta$–estradiol and estrone levels in postmenopausal women with and without endometrial cancer. *J. Clin. Endocrinol. Metab.*, **43**, 272.

KELSEY, J.L., LiVOLSI, V.A., HOLFORD, T.R. & 5 others (1982). A case-control study of cancer of the endometrium. *Am. J. Epidemiol.*, **116**, 333.

LA VECCHIA, C., FRANCESCHI, S., DECARLI, A., GALLUS, G. & TOGNONI, G. (1984). Risk factors for endometrial cancer at different ages. *J. Natl Cancer Inst.*, **73**, 667.

LAWRENCE, C., TESSARO, I., DURGERIAN, S. & 4 others (1987). Smoking, body weight, and early-stage endometrial cancer. *Cancer*, **59**, 1665.

LESKO, S.M., ROSENBERG, L., KAUFMAN, D.W. & 8 others (1985). Cigarette smoking and the risk of endometrial cancer. *N. Engl. J. Med.*, **313**, 593.

LEVI, F., LA VECCHIA, C. & DECARLI, A. (1987). Cigarette smoking and the risk of endometrial cancer. *Eur. J. Cancer Clin. Oncol.*, **23**, 1025.

MACK, T.M., PIKE, M.C., HENDERSON, B.E. & 4 others (1976). Estrogens and endometrial cancer in a retirement community. *N. Engl. J. Med.*, **294**, 1262.

MacMAHON, B., TRICHOPOULOS, D., COLE, P. & BROWN, J. (1982). Cigarette smoking and urinary estrogens. *N. Engl. J. Med.*, **307**, 1062.

McDONALD, T.W., ANNEGERS, J.F., O'FALLON, W.M., DOCKERTY, M.B., MALKASIAN, G.D. & KURLAND, L.T. (1977). Exogenous estrogen and endometrial carcinoma: case-control and incidence study. *Am. J. Obstet. Gynecol.*, **127**, 572.

MICHNOVICZ, J.J., HERSHCOPF, R.J., NAGANUMA, H., BRADLOW, H.L. & FISHMAN, J. (1986). Increased 2–hydroxylation of estradiol as a possible mechanism for the anti-estrogenic effect of cigarette smoking. *N. Engl. J. Med.*, **315**, 1305.

MOOLGAVKAR, S.H., DAY, N.E. & STEVENS, R.G. (1980). Two-stage model for carcinogenesis: epidemiology of breast cancer in females. *J. Natl Cancer Inst.*, **65**, 559.

MOORE, J.W., CLARK, G.M.G., TAKATANI, O., WAKABAYASHI, Y., HAYWARD, J.L. & BULBROOK, R.D. (1983). Distribution of 17$\beta$-estradiol in the sera of normal British and Japanese women. *J. Natl Cancer Inst.*, **71**, 749.

PARDRIDGE, W.M. (1986). Serum bioavailability of sex steroid hormones. *Clinics Endocrinol. Metabol.*, **15**, 259.

PIKE, M.C., KRAILO, M.D., HENDERSON, B.E., CASAGRANDE, J.T. & HOEL, D.G. (1983). 'Hormonal' risk factors, 'breast tissue age' and the age-incidence of breast cancer. *Nature*, **303**, 767.

PIKE, M.C. (1987). Age-related factors in cancers of the breast, ovary and endometrium. *J. Chron. Dis.*, **40**, Suppl II, 59.

ROGERS, J. & MITCHELL, G.W. (1952). The relation of obesity to menstrual disturbances. *N. Engl. J. Med.*, **247**, 53.

SHAPIRO, S., KELLY, J.P., ROSENBERG, L. & 7 others (1985). Risk of localized and widespread endometrial cancer in relation to recent and discontinued use of conjugated estrogens. *N. Engl. J. Med.*, **313**, 969.

SHERMAN, B.M. & KORENMAN, S.G. (1974). Measurement of serum LH, FSH, estradiol and progesterone in disorders of the human menstrual cycle: the inadequate luteal phase. *J. Clin. Endocrinol. Metabol.*, **39**, 145.

SIITERI, P.K. (1978). Steroid hormones and endometrial cancer. *Cancer Res.*, **38**, 4360.

SIITERI, P.K. & MacDONALD, P.C. (1973). Role of extraglandular estrogen in human endocrinology. In *Handbook of Physiology*, Geiger *et al.* (eds) p. 615. American Physiological Society: Washington DC.

STUDD, J.W.W., THOM, M.H., PATERSON, M.E.L. & WADE-EVANS, T. (1980). The prevention and treatment of endometrial pathology in postmenopausal women receiving exogenous oestrogens. In *The Menopause and Postmenopause*, Pasetto, N. *et al.* (eds) p. 127. MTP Press: Lancaster.

THORNEYCROFT, I.H., MISHELL, D.R., STONE, S.C., KHARMA, K.M. & NAKAMURA, R.M. (1971). The relation of serum 17-hydroxyprogesterone and estradiol-17$\beta$ levels during the human menstrual cycle. *Am. J. Obstet. Gynecol.*, **111**, 947.

TYLER, C.W., WEBSTER, L.A., ORY, H.W. & RUBIN. G.L. (1985). Endometrial cancer: how does cigarette smoking influence the risk of women under age 55 years having this tumor? *Am. J. Obstet. Gynecol.*, **151**, 899.

WEISS, N.S., SZEKELY, D.R., ENGLISH, D.R. & SCHWEID, A.I. (1979). Endometrial cancer in relation to patterns of menopausal estrogen use. *J. Am. Med. Assoc.*, **242**, 261.

WEISS, N.S., FAREWELL, V.T., SZEKELY, D.R., ENGLISH, D.R. & KIVIAT, N. (1980). Oestrogens and endometrial cancer: effect of other risk factors on the association. *Maturitas*, **2**, 185.

WHITEHEAD, M.I. (1978). The effects of oestrogens and progestogens on the postmenopausal endometrium. *Maturitas*, **1**, 87.

WHITEHEAD, M.I., TOWNSEND, P.T., PRYSE-DAVIES, J., RYDER, T.A. & KING, R.J.B. (1981). Effects of estrogens and progestins on the biochemistry and morphology of the postmenopausal endometrium. *N. Engl. J. Med.*, **305**, 1599.

WHITEHEAD, M.I., TOWNSEND, P.T., PRYSE-DAVIES, J. & 4 others (1982). Effects of various types and dosages of progestogens on the postmenopausal endometrium. *J. Reprod. Med.*, **27**, 539.

WILLETT, W.C., BROWNE, M.L., BAIN, C. & 6 others (1985). Relative weight and risk of breast cancer among premenopausal women. *Am. J. Epidemiol.*, **122**, 731.

ZUMOFF, B. (1982). Relationship of obesity to blood estrogens. *Cancer Res.* (*Suppl.*), **42**, 3289s.